# Bacterial Population in Intestines of the Black Tiger Shrimp (*Penaeus monodon*) under Different Growth Stages

**DOI:** 10.1371/journal.pone.0060802

**Published:** 2013-04-05

**Authors:** Wanilada Rungrassamee, Amornpan Klanchui, Sage Chaiyapechara, Sawarot Maibunkaew, Sithichoke Tangphatsornruang, Pikul Jiravanichpaisal, Nitsara Karoonuthaisiri

**Affiliations:** 1 Microarray Laboratory, National Center for Genetic Engineering and Biotechnology, Khlong Luang, Pathum Thani, Thailand; 2 Aquatic Molecular Genetics and Biotechnology Laboratory, National Center for Genetic Engineering and Biotechnology, Khlong Luang, Pathum Thani, Thailand; 3 Genomic Research Laboratory, National Center for Genetic Engineering and Biotechnology, Khlong Luang, Pathum Thani, Thailand; Charité-University Medicine Berlin, Germany

## Abstract

Intestinal bacterial communities in aquaculture have been drawn to attention due to potential benefit to their hosts. To identify core intestinal bacteria in the black tiger shrimp (*Penaeus monodon*), bacterial populations of disease-free shrimp were characterized from intestines of four developmental stages (15-day-old post larvae (PL15), 1- (J1), 2- (J2), and 3-month-old (J3) juveniles) using pyrosequencing, real-time PCR and denaturing gradient gel electrophoresis (DGGE) approaches. A total of 25,121 pyrosequencing reads (reading length = 442±24 bases) were obtained, which were categorized by barcode for PL15 (7,045 sequences), J1 (3,055 sequences), J2 (13,130 sequences) and J3 (1,890 sequences). Bacteria in the phyla *Bacteroides*, *Firmicutes* and *Proteobacteria* were found in intestines at all four growth stages. There were 88, 14, 27, and 20 bacterial genera associated with the intestinal tract of PL15, J1, J2 and J3, respectively. Pyrosequencing analysis revealed that *Proteobacteria* (class *Gammaproteobacteria*) was a dominant bacteria group with a relative abundance of 89% for PL15 and 99% for J1, J2 and J3. Real-time PCR assay also confirmed that *Gammaproteobacteria* had the highest relative abundance in intestines from all growth stages. Intestinal bacterial communities from the three juvenile stages were more similar to each other than that of the PL shrimp based on PCA analyses of pyrosequencing results and their DGGE profiles. This study provides descriptive bacterial communities associated to the black tiger shrimp intestines during these growth development stages in rearing facilities.

## Introduction

The intestinal tract is a complex ecosystem that harbors a diverse bacterial community and this bacterial population has been shown to have profound influence on immunity, nutrient processing and protective processes in the host animal [1], [2], [3], [4], [5], [6]. In the zebrafish model, the gut microbial community plays a significant role in digestive tract development [7]. The colonization of microorganisms in germfree zebrafish modulates expression of host genes involved in nutrient absorption, mucosal modification and immunity [3]. Bacteria in intestines of other aquatic animals have also been shown to contribute to host’s immune system development, and the potential application of beneficial bacteria to aquaculture feeds, especially in fish and shellfish larviculture, has been increasingly investigated [8]. However, the knowledge about this bacterial community in these aquatic species is still limited. Thus, such an investigation will help shedding light on beneficial bacteria that help maintaining the health of domestic animal stocks [5].

The black tiger shrimp (*Penaeus monodon*) is a prominent marine shrimp with high economic value in Southeast Asia, the Sea of Japan and Australia regions [9], [10]. Commercial production of the black tiger shrimp has been in a major decline due to higher incidents of viral and bacterial disease outbreaks [10], [11]. Many studies in crustaceans, including the black tiger shrimp, focused on the development of probiotic applications to enhance disease resistance and growth [12], [13], [14]. However, a probiotic approach for the black tiger shrimp is challenging due to the lack of understanding of natural microflora and factors contributing to the diversity of the bacterial population. The life cycle of penaeid shrimp includes egg, larval (nauplius, protozea and mysis), post-larval, juvenile and adult stages. Shrimp from the post-larval through juvenile stages have been reported to have higher bacterial disease susceptibility than those at the later stages [15], [16], [17], [18]. Being aquatic species, shrimp are constantly exposed to a variety of bacteria and viruses, which some can be pathogenic. Shrimp pathogens can orally enter, invade digestive tract system and cause infection [19]. Although shrimp has innate immune system to battle against the pathogen’s invasion, it has been speculated that bacterial community in shrimp intestines may play protective roles as natural barriers. Thus far, bacteria associated with the intestine of black tiger shrimp at the early developmental stages have not yet been characterized.

Traditionally, bacterial populations in the intestines have been characterized by both culture-dependent and culture-independent techniques based on 16S rRNA sequences [20], [21], [22]. The 16S rRNA sequences from these approaches are then individually sequenced using the Sanger sequencing method. Thus, the limited number of clones or DNA bands selected for sequencing might not give a complete picture to represent actual bacterial ecology. To provide comprehensive coverage of bacterial communities, a high-throughput pyrosequencing method has been employed to obtain a higher number of sequence-reads [23]. The method has recently gained popularity for an in-depth characterization of various microbial communities such as those in the human [24], [25], [26], fermented food [27], soil [28], [29], [30], [31], extreme environments [32], the deep marine biosphere [33], [34], wastewater treatment bioreactors, [35] and plant biospheres [36], [37]. Furthermore, to enable an analysis of multiple samples in parallel within a single run, a barcode tag with a short unique nucleotide sequence was added to distinguish each sample [31].

This study provides the first report on intestinal bacterial populations in the black tiger shrimp at different growth stages using pyrosequencing approach and the results were validated by additional methods of real-time PCR and denaturing gradient gel electrophoresis (DGGE). The rearing of black tiger shrimp from post-larval to juvenile stages encounters high risk of bacterial disease outbreak. In this work, we aim to identify bacterial populations that provide natural barriers to host shrimp in rearing facilities by characterization of bacterial communities in intestines of 15-day-old post larval, 1-, 2- and 3-month-old juvenile stages. The bacterial populations from different growth stages were also compared to identify core bacteria-associated to the black tiger shrimp intestines throughout growth development in rearing facilities.

## Results and Discussion

The microbiota and their influences on immune response, disease resistance, and nutrition for the animal hosts have been intensively studied in many vertebrate model organisms [3], [38], [39]. However, less is known about the intestinal bacterial community in other non-model organisms such as the black tiger shrimp. This study provides the first report on bacteria associated in intestines of the black tiger shrimp by following four different growth stages of the same shrimp population. Three culture-independent approaches, pyrosequencing, real-time PCR and denaturing gradient gel electrophoresis (DGGE), were employed to characterize bacterial diversity and community structure in shrimp intestines.

To determine bacterial populations, pyrosequencing of the 16S rRNA gene amplicons were performed in intestine samples of 15-day-old post larval (PL15), 1- (J1), 2- (J2) and 3-month-old (J3) juvenile shrimp. A total of 25,121 sequences of V3-6 regions of the 16S rRNA gene were obtained from the barcoded pyrosequencing method and with an average read length of 442±24 bases (Table 1). All sequences were sorted by a barcode for each library: PL15 (7,045 sequences), J1 (3,055 sequences), J2 (13,130 sequences) and J3 (1,890 sequences). Sequences were clustered into operational taxonomic units (OTUs) at 0.03 dissimilarity levels, where each OTU represents unique phylotype. The total number of OTUs was the highest in PL15 (514 OTUs), where J1, J2 and J3 contained 97, 298, and 167 OTUs, respectively. Interestingly, number of OTUs decreased as shrimp were developed to juvenile stage. When sequences were classified into taxonomic ranks using the Ribosomal Database Project (RDP) Bayesian classifier with a confidence threshold of 80% [40], phylum distribution of bacteria in intestines at four growth stages varied from 3 to 6 phyla. The intestines of PL15 showed the highest diversity of bacteria (88 genera), where J1, J2 and J3 had 14, 27 and 20 genera, respectively (Table 1).

**Table 1:** Sampling depth and biodiversity analysis of 16S rRNA sequences from barcoded pyrosequencing of the black tiger shrimp intestines from different growth stages.

	Growth stage^1^			
	PL15	J1	J2	J3
**Sampling depth**				
A total number of sequences (25,121)	7,045	3,055	13,130	1,890
OTUs (0.03 dissimilarity level)	514	97	298	167
Phylum	6	4	6	3
Class	12	8	10	4
Family	52	15	24	14
Genus	88	14	27	20
**Diversity indices**				
Good’s Coverage	0.96	0.98	0.98	0.94
Shannon	3.46	0.58	0.67	2.86
Chao1	1,016	277	896	482

1 PL15 denotes 15-day-old post lava, whereas J1, J2 and J3 denote 1-, 2- and 3-month-old juvenile, respectively.

To estimate and compare bacterial diversity among growth stages, the OTUs from each library were used to calculate three diversity indices: Good’s coverage, Shannon and Chao1 (Table 1). Good’s coverage index estimates the percentage of total bacterial OTUs represented in a sample, in which the four growth stage libraries had the values in a range of 0.94–0.98 (Table 1). When its value approaches 1.0, it indicates that the obtained sequences from each library represent the majority of bacterial sequences in the sample [41]. Bacterial diversity was estimated by Shannon index, in which a higher value means a greater bacterial diversity [42]. In this work, PL15 library had higher bacterial diversity (Shannon index = 3.46) than that of J1, J2 and J3 libraries (0.58, 0.67, and 2.86, respectively). Due to the lack of pyrosequencing data of bacterial diversity in invertebrates, especially in aquatic organisms, we compared our results to well-studied model animals, in which bacteria from juvenile intestines (Shannon index at 0.58–2.86) show much lower diversity than those from humans (4.0) [43], [44], and mouse (5.5) [45]. Different host animal digestive systems and habitats, such as aquatic and terrestrial habitats, might contribute to different levels of bacterial diversity. Lower bacterial diversity might suggest that the habitat within the shrimp intestine was more dynamic with harsher conditions than mammalian hosts, thus, fewer bacteria can adapt under such environments.

To estimate true bacterial species richness, Chao1 index was calculated and its value reflects an estimated OTU for each sample [46]. Chao1 values were calculated in PL15 (1,016), J1 (277), J2 (896) and J3 (482). All Chao1 values were higher than the observed OTUs in all growth stages, indicating that more bacterial species are still expected in shrimp intestines. In consistent to Chao1 analysis, rarefraction curves calculated at a distance of 0.03 did not reach saturation for the four growth stages even in the J2 library that contained >10,000 reads (Fig. S1). Both Chao1 and rarefraction curve analyses indicate that bacterial richness in the black tiger shrimp intestines are expected to be greater than described in this study. The estimation of bacterial richness is a major challenge in various ecologies. For instance, rarefraction curve analysis of bacterial communities in zebrafish intestines has not been saturated, even when a range of 5,000 to 10,000 sequence reads were obtained [47].

**Figure 1:**
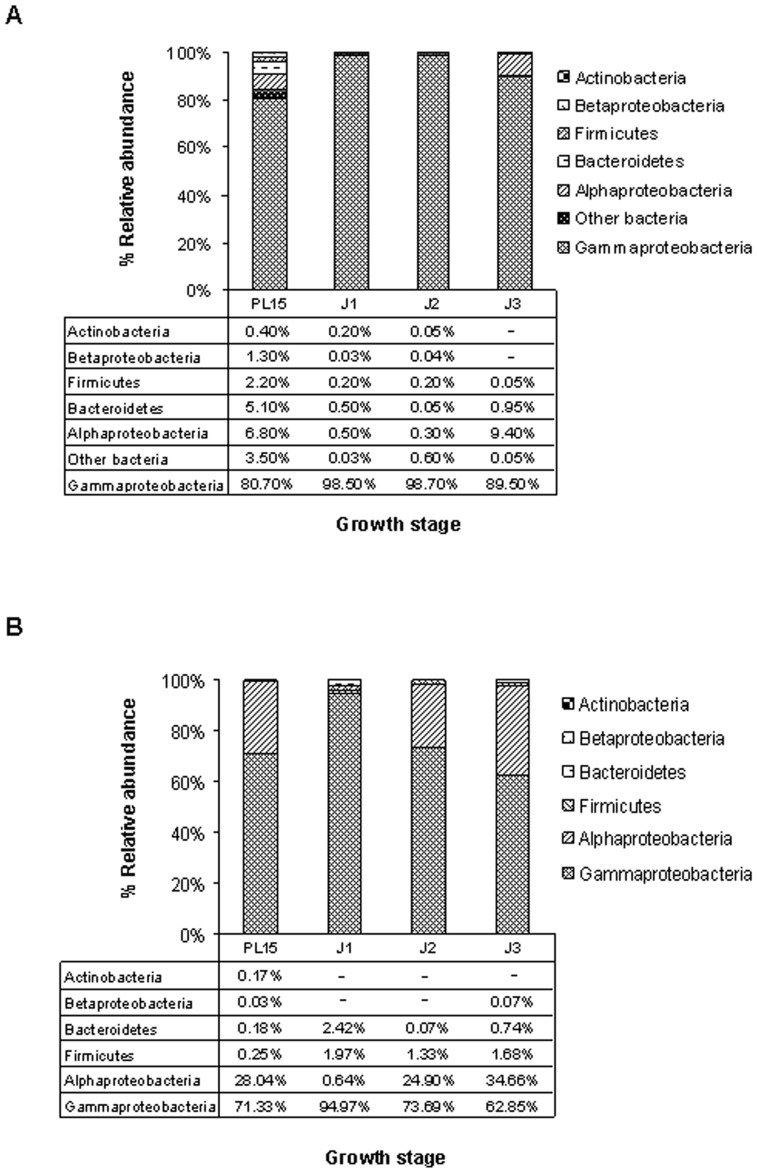
Frequency distribution of phylogenetic groups in intestines of different growth stages of the black tiger shrimp: 15-day-old post-larva (PL15) and 1-, 2- and 3-month-old juveniles (J1, J2 and J3, respectively). (A) Percent distribution of bacterial phylum by pyrosequencing analysis and (B) relative abundance of the six bacterial phylogenetic groups estimated by real-time PCR.

Bacterial pyrosequencing reads were classified to phyla *Actinobacteria*, *Firmicutes*, *Bacteroidetes, Proteobacteria (*class: *Alpha-, Beta-* and *Gammaproteobacteria*) (Fig. 1 and Table S1). *Fusobacteria*, *Spirochaetes* and unclassified bacteria were grouped as other bacteria (Fig. 1A). Of these, *Gammaproteobacteria* (phylum *Proteobacteria*) were the most abundant, accounting for more than 80% in shrimp intestines from all growth stages (Fig. 1A). In addition to *Proteobacteria*, *Bacteroidetes* and *Firmicutes* were also found in all growth stages (Fig. 1A), suggesting that these phyla constituted as core intestinal bacteria in the black tiger shrimp. Bacteria in these phyla are also commonly found associated in intestines of many other aquatic species [20], [48], [49]. In attempt to identify core intrinsic bacterial groups in the black tiger shrimp’s intestine regardless of rearing environment, bacterial community analysis in intestines of wild-caught black tiger shrimp is underway and results will be compared to this study to identify shared core bacterial members.

To validate bacterial abundance from pyrosequencing results, bacterial groups in shrimp intestines were further analyzed by real-time PCR at the higher taxonomic levels (Fig. 1B). Bacteria were detected in each sample using specific primers for *Firmicutes*, *Alpha-, Beta*- and *Gammaproteobacteria*, *Actinobacteria* and *Bacteroidetes*. From the real-time PCR result, the obtained relative abundance revealed that bacteria from class *Gammaproteobacteria* were dominant in the shrimp intestines from all growth stages, which was consistent with the pyrosequencing observation. The previous survey of bacterial population in the black tiger shrimp juveniles from the commercial farms also shows that *Gammaproteobacteria* was predominated in intestines [20].

**Figure 2:**
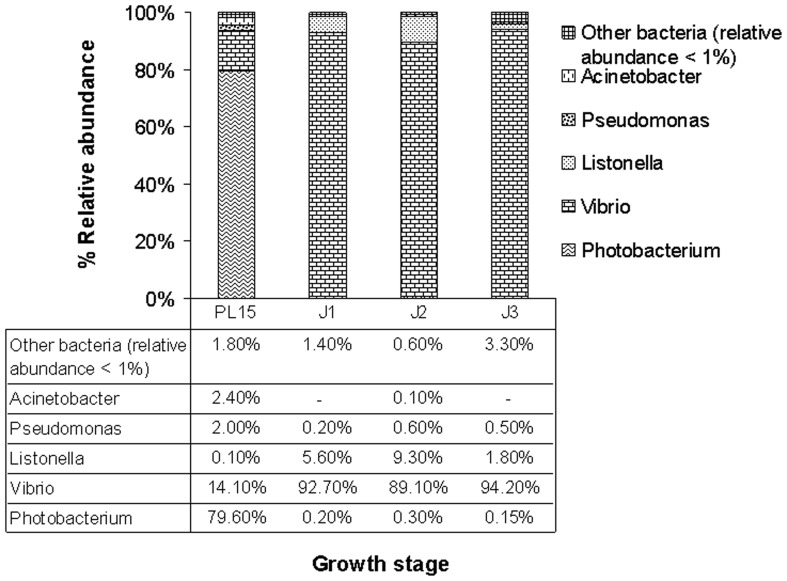
Frequency distribution of selected genera with high abundance in *Gammaproteobacteria* from pyrosequencing analysis. PL15 denotes 15-day-old post-larva (PL15) whereas J1, J2 and J3 denote 1-, 2- and 3-month-old juveniles, respectively.

Within class *Gammaproteobacteria,* the main bacterial genera were from the family *Vibrionaceae* (Fig. 2 and Table S2). This family of bacteria are widely distributed in marine environment [50] and has previously been reported to be found in high relative abundance in intestine of various aquatic marine organisms including the black tiger shrimp [21], [22], [48], [49], [51]. *Photobacterium* species were the majority during the post-larval stage (80%) and then the dominant bacterial group shifted to *Vibrio* species during the juvenile stages (Fig. 2). Other less abundant bacteria were found sporadically. *Fusobacteria* were only found in the PL15 intestines, whereas *Spirochaetes* were only associated with J2 intestines. *Actinobacteria* were found in both PL15 and J2 intestines. Moreover, *Listonella* species were found only in the intestines of the juvenile stages, while the genera *Labrenzia* and *Silicibacter* were found only in intestines of the J3 stage.

The difference in bacterial composition in post-larva and juveniles might also be due to different feed conditions. In this study, bacterial populations were investigated in shrimp rearing under commercial production, where post-larval shrimp were fed with live *Artemia* nauplii (brine shrimp) as nutritional supplement in addition to commercial feed pellets until they reach juvenile stage. Once shrimp reached juvenile stages, their diet is solely based on commercial pellets [52], [53]. The gut layer in Penaeid shrimp is ectodermal origin, which regularly shreds off during the molting period [54]. Therefore, bacteria from ingested food influence re-colonization of the shrimp gut’s microbial flora after shredding. The shifting from the live feed in post-larval shrimp to commercial pellets in juvenile shrimp might explain the difference in diversity of bacterial composition in shrimp rearing ponds because bacteria from *Proteobacteria*, *Bacteroidetes*, *Firmicutes*, *Actinobacteria* have also been found associated to *Artemia* nauplii [55]. Moreover, several studies have shown that *Artemia nauplii* are one of major vectors to introduce bacteria including pathogens to hatchery system [56], [57].

**Figure 3:**
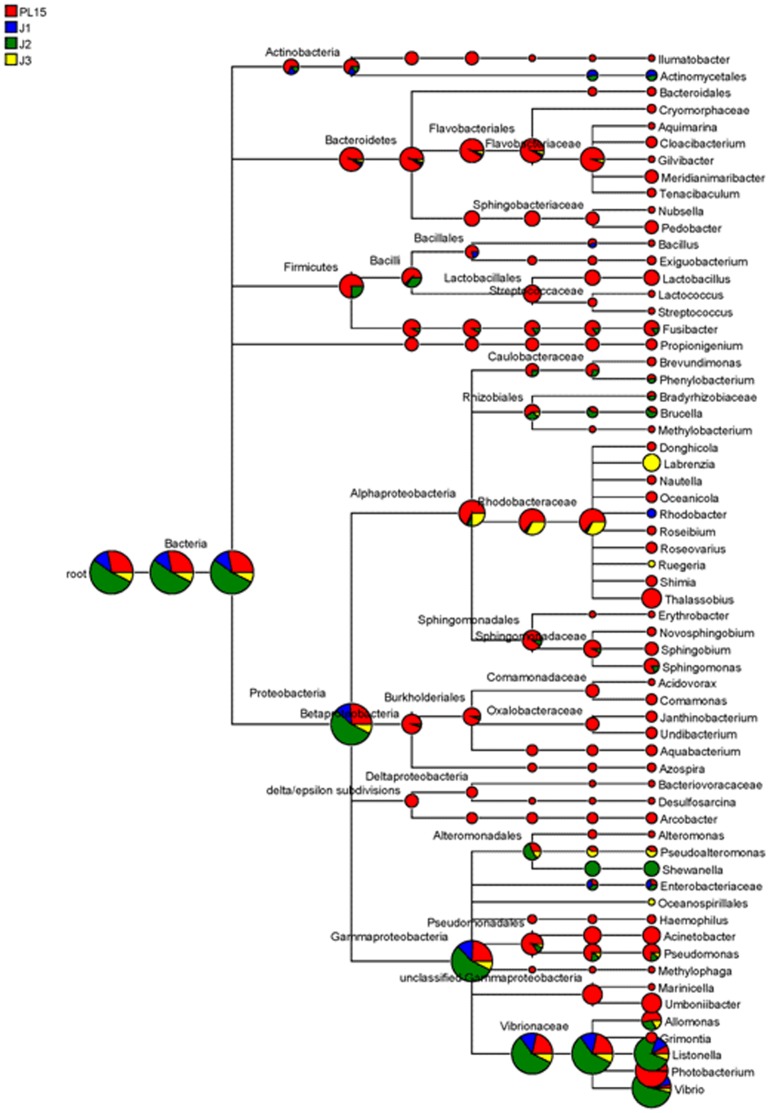
Comparison of the bacterial compositions in shrimp intestines of four growth stages: 15-day-old post-larva (PL15) and 1-, 2- and 3-month-old juveniles (J1, J2 and J3, respectively). Pie charts of the hierarchical tree reflect relative abundance for each genus from each library (red represents PL15, blue represents J1, green represents J2 and yellow represents J3).

**Figure 4:**
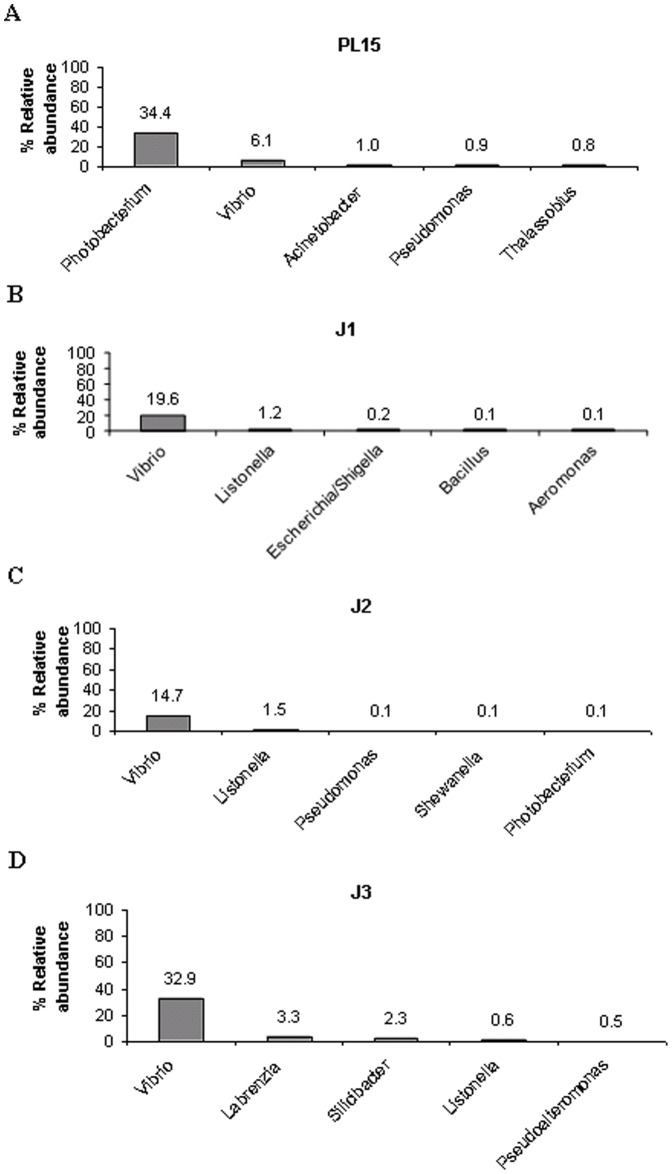
Top five most abundant bacterial genera in shrimp intestines of four growth stages: A) 15-day-old post-larva (PL15), B) 1-month-old juveniles (J1), C) 2-month-old juveniles (J2) and D) 3-month-old juveniles (J3).

To visualize bacterial composition in hierarchical orders, the classified pyrosequencing reads from all four growth stages were used to construct pie charts to represent relative abundance for each taxonomic node (Fig. 3) and the observed frequency of pyrosequencing reads in each library was shown in Table S1. The pyrosequencing reads that could not be classified at genus level were classified to the nearest match of the next taxonomic ranking (Table S1). These unclassified sequences suggest that many bacteria-associated with shrimp intestine were still uncultured or might not be previously reported. The bacterial populations of PL15 were highly diverse and found in most of the taxonomic nodes, whereas those bacteria found in juvenile stages were more closely related (Fig. 3). The top five most abundant bacteria genera were compared among the four libraries (Fig. 4). In the PL15 library, *Photobacterium* (34.4%) was the highest, followed by *Vibrio* (6.1%), *Acinetobacter* (1.0%), *Pseudomonas* (0.9%) and *Thalassobius* (0.8%) (Fig. 4A). The J1 and J2 libraries contained similar dominant genera: *Vibrio* (19.6% and 14.7%, respectively) and *Listonella* (1.2% and 1.5%, respectively) (Fig. 4B and C). However, the J1 and J2 libraries differ in the minor bacteria. The J1 had *Escherichia/Shigella* (0.2%), *Bacillus* (0.1%) and *Aeromonas* (0.1%), while the J2 contained 0.1% of *Pseudomonas, Shewanella* and *Photobacterium.* In the J3 library, the bacterial population was 32.9% *Vibrio*, 3.3% *Labrenzia*, 2.3% *Silicibacter*, 0.6% *Listonella* and 0.5% *Pseudoalteromonas* (Fig. 4D). The bacterial genera shared among the four growth stages were *Vibrio*, *Photobacterium*, *Pseudomonas*, and *Listonella*.

**Figure 5:**
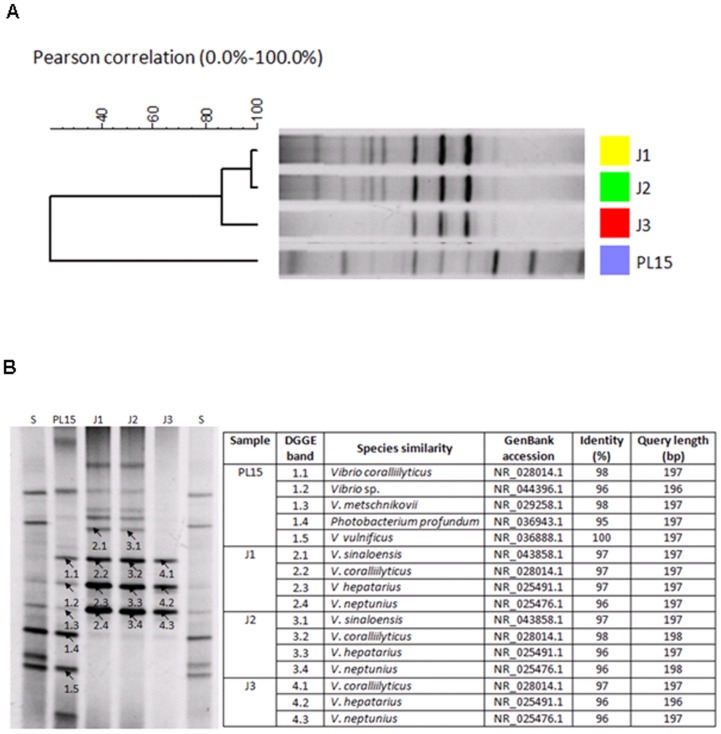
PCR-DGGE analysis of the predominant bacterial population in intestines of black tiger shrimp from different growth stages: 15-day-old post-larva (PL15), 1-, 2- and 3-month-old juveniles (J1, J2 and J3, respectively) and S is in-house standard marker. (A)Dendogram analysis of DGGE profile and (B) bacterial profiles and species similarity of selected DGGE bands.

**Figure 6:**
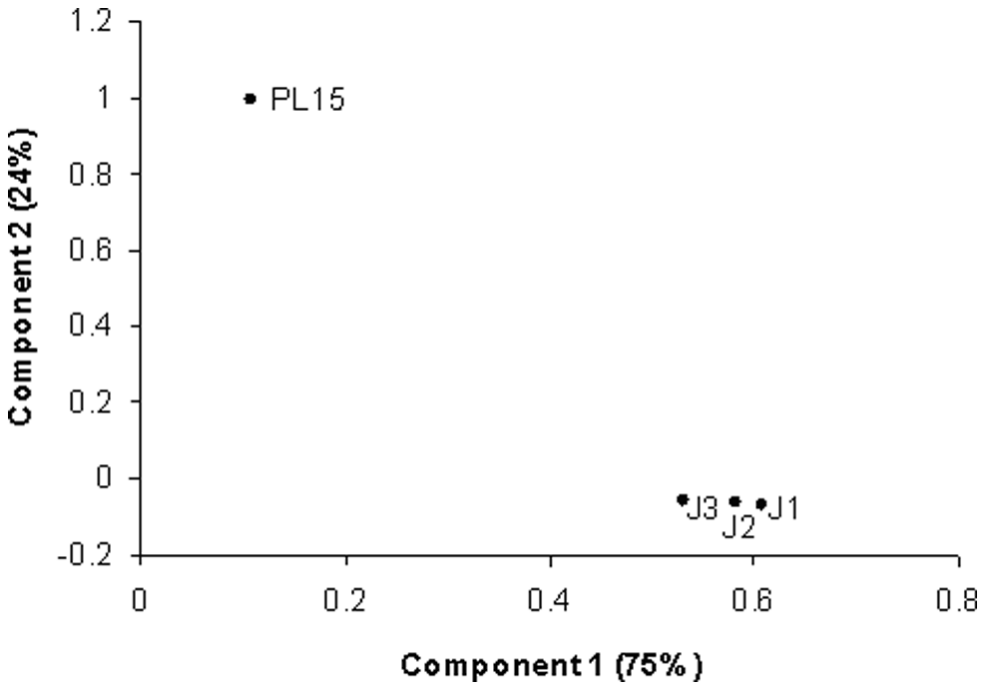
Principal component analysis of bacterial populations in intestines of different growth stages of the black tiger shrimp: 15-day-old post-larva (PL15) and 1-, 2- and 3-month-old juveniles (J1, J2 and J3, respectively). The principal component analysis (PCA) was performed using R script to compare bacterial community structures among the samples based on relative abundance of various bacteria genera.

In addition to pyrosequencing and real-time PCR, bacterial profiles in the four growth stages were also compared using DGGE analysis (Fig. 5). The DGGE profiles were clustered according to Pearson correlation, and it showed two distinct groups, PL15 and the juvenile stages (Fig. 5A). DGGE profiles of J1, J2 and J3 were at 85% similarity level. Sequences from the dominant DGGE bands were similar to those of *Vibrio* species in all four stages, and *Photobacterium* species was detected in PL15 (Fig. 5B). Principal component analysis based on relative bacterial abundance from pyrosequencing results showed the similar trend to DGGE profile analysis, in which the bacterial profile from PL15 intestines was in a different cluster from those found in the juvenile stages (Fig. 6). Although our DGGE analysis revealed only bacteria genera with high abundance and may not provide an in-depth analysis of bacterial population, this method is proven to be useful for comparison of bacteria community profiles in food and soil samples [27], [58], [59]. Our study also showed that bacterial patterns from DGGE and pyrosequencing were congruence. The DGGE analysis can therefore be used as a validation technique to confirm the results from the pyrosequencing analysis, which can detect bacterial genera with low abundance in a sample.

One other factor that influences on intestinal bacterial diversity is the selective pressure from the host such as resistance to host immune system [60]. The shrimp digestive system is fully developed in the post-larval stage [61]. However, the immune system of post-larval shrimp is not as developed as in juvenile shrimp [19], [62]. For example, penaedin, a shrimp antibacterial peptide, is expressed weakly in post-larval shrimp in comparison to juvenile shrimp [62]. This factor, in addition to the difference in shrimp diets between different developmental stages might contribute to a higher bacterial diversity post-larval shrimp [5], [63]. The lower immune level in the post-larval stage resulted in less harsh condition for bacteria to reside within [60], [62], resulting in different intestinal bacterial diversity from the harsher conditions in the juveniles. In contrast, the more developed immune system in juvenile shrimp imposes a higher selective pressure for those bacteria that can resist the host immune system, resulting in more closely related bacteria found in their intestines. The intrinsic developmental changes in transition to later growth stage may exert selective pressure for those highly adapted bacteria to host gut habitat [64], [65]. Moreover, the digestive tract of a juvenile shrimp has more surface area than that of the post-larvae, and this might allow establishment of resident bacterial population.

In this study, *Bacteroidetes*, *Firmicutes* and *Proteobacteria* were core phyla found in intestines of post-larvae and juvenile shrimp and selecting bacterial candidates from resident population might be another mean for successful probiotics development. Bacteria associated with animal digestive tract might be beneficial to the host such as natural barriers against pathogen colonization through competition for binding sites [66]. Our findings showed that *Proteobacteria* (class *Gammaproteobacteria)*, particularly *Vibrio* species dominated the intestines at all three juvenile stages, and *Photobacterium* species was the highest in intestines at the post-larval stage. While some *Vibrio* species are reported as shrimp pathogens [17], [67], there is some evidence suggesting beneficial roles of *Vibrio* species to increase disease resistance in aquatic animal hosts [21]. Additionally, many studies have focused on the search for beneficial bacteria for marine shrimp farming, and the indigenous bacteria found in shrimp intestines may be more effective for probiotic candidates due to their abilities to survive and colonize inside host shrimp. The main bacterial groups that have been tested as probiotics in aqua-feeds are *Vibrio*, *Pseudomonas*, *Bacillus* and *Lactobacillus* [13], [68], and they were present in our survey of bacteria populations in intestines of post-larval and juvenile stages. Although intestinal bacterial population of shrimp in their early stage was highly diverse, we observed that they became more progressively similar in juvenile stages. Understanding the relationship between intestinal bacterial communities and their influence on the health and survival of their hosts under bacterial pathogen invasion is essential. In the case of opportunistic pathogens, if intestinal bacterial balance is maintained, the risk of disease outbreak in shrimp farms may be reduced. Bacterial population dynamics in the presence of shrimp pathogens in the black tiger shrimp intestines will be further investigated. Approaches to maintain intestinal bacterial balance especially in early life stage will contribute to lower disease outbreak in shrimp rearing facilities. Identification of the intestinal microflora in forming a protective barrier and out-competing pathogenic bacteria in the black tiger shrimp will benefit the shrimp industry.

## Materials and Methods

### Animals and Intestine Sample Collection

The black tiger shrimp (*P. monodon*) were hatched at the Shrimp Genetic Improvement Center (Surat Thani province, Thailand) and routinely checked to ensure that they are free from specific-pathogens: Taura Syndrome Virus, White Spot Syndrome Virus, Yellow Head Virus, Monodon Baculovirus, and Infectious Hypodermal and Hematopoietic Necrosis Virus [10]. Shrimp larvae were reared in cement tanks at 15 ppt salinity and fed 4 times daily with *Artemia* nauplii and commercial feed with high protein formula (Starfeeds for PL containing 50% proteins, CPF, Thailand). Once the larvae were 15-days old, they were transferred to a plastic-lined pond (20×20 m) at 10–11 ppt salinity and fed 4 times daily with commercial feed (Starfeeds containing 40% proteins) at the Marine Technology Research Center, Faculty of Marine Technology (Burapha University Chanthaburi Campus, Thailand). Shrimp at 15-day of post-larval, 1-, 2- and 3-month-old juvenile stages (PL15, J1, J2 and J3, respectively) were collected 2 hours after the morning feeding. Intestines from post-larval stages were aseptically dissected using sterile needles under a dissecting microscope, and intestines from juvenile stages were dissected from the shrimp using sterile surgical blades and kept frozen in liquid nitrogen. All intestine samples were stored at −80°C until use.

### DNA Extraction and Purification

The intestine samples were collected from the same shrimp family, in which all of them were reared under the same environment conditions. Our previous study shows that intestinal bacteria from individual shrimp rearing under the same environmental conditions exhibit similar bacterial profile [20]. Therefore, to obtain high efficiency of DNA extraction, 5 intestine samples were pooled for juvenile shrimp. Due to relatively small mass of PL15 intestine to those of juveniles, 50 intestines were pooled in order to obtain a comparable total mass of intestines to juvenile intestines and to obtain adequate mass for subsequent experiments. Genomic DNA was extracted using the QIAamp DNA mini kit (Qiagen, Germany) according to the manufacturer’s instructions. The DNA purity and concentration was determined by the NanoDrop (ND-8000) spectrophotometer and DNA samples were kept at −20°C for subsequent analyses.

### Pyrosequencing of Barcoded 16S rRNA Genes and Data Analysis

To prepare DNA libraries for barcoded pyrosequencing, the V3-6 region of 16S rRNA gene was amplified with the 338F and 786R primer pair using high-fidelity DNA polymerase (Novagen, Germany). The primers were modified with a barcode tag by adding a sequence of 8 nucleotides to the 5′ end of each primer pair (Table S2, underlined sequence). The PCR products were gel purified with the Qiaquick Gel Extraction kit (Qiagen), ligated to 454-adapters (Table S2) and sequenced on the Genome Sequencer Junior platform according to Roche protocol (454 GS Junior System, Roche, USA).

The sequence data was processed using Mothur software to remove low quality sequences [69]. In Mothur, poor quality sequences were set as sequences with a length less than 200 bases, contained ambiguous bases and homopolymers greater than 6 bases or did not have a barcode and a primer sequence. All sequences were checked for chimeras by UCHIME [70]. The sequence data were analyzed by the RDP II Classifier with an 80% confidence threshold [40]. Operational taxonomy units (OTUs) were clustered by Mothur using the average neighbor algorithm at 0.03 dissimilarity level to classify to OTUs [71]. Shannon index and Chao1 biodiversity index values were calculated for each shrimp library using Mothur. The coverage index was calculated by 1– (n/N), where n is the number of phylotypes and N is the total number of sequences [41]. Only pyrosequencing reads with minimum frequency of five were used to construct a hierarchical tree to visualize and compare relative abundance of each genus from all growth stages. The taxonomic tree was constructed using MEGAN based on the results of taxonomical assignment by the RDP classifier [72].

Principal component analysis was performed using R script to compare bacterial community structures among the samples based on relative abundance of various bacteria genera.

All nucleotide sequence data in this study were deposited in GenBank database with accession numbers JX919344 to JX926388 for the PL15 library, JX916289 to JX919343 for the J1 library, JX926389 to JX939518 for the J2 library and JX939519 to JX941408 for the J3 library.

### Real-time PCR Analysis of Bacterial Phyla

To validate pyrosequencing results, specific bacterial groups were determined by real-time PCR. Detection of bacterial taxa by real-time PCR assay and primer specificity was previously developed [73], [74]. Primer pairs for all bacteria, *Firmicutes*, *Betaproteobacteria*, *Gammaproteobacteria*, *Alphaproteobacteria*, *Actinobacteria* and *Bacteroidetes* were listed in Table S3. For each intestinal DNA sample, the real-time PCR were carried out in triplicates with IQ SYBR Green supermix (Bio-Rad Laboratories, Inc., USA) in CFX96 real-time detection system (Bio-Rad Laboratories, Inc.). Each 20-µl reaction contained 100 ng of DNA template, 200 nM of each primer, 10 µl of IQ SYBR green supermix and sterile water to complete the final 20 µl volume. The real-time cycle parameters were initial denaturation at 95°C for 3 min, 40 cycles of denaturation at 95°C for 30 sec, annealing at 54°C for 20 sec and extension at 72°C for 30 sec, fluorescent signal was detected at the end of each cycle. Melting curve analysis was performed from 55°C to 95°C with continuous fluorescence reading every 0.5°C increment to validate specificity of PCR amplification. The relative folds of bacterial abundance were calculated as previously described [73]. Briefly, a plasmid DNA containing target sequence was constructed from PCR amplification using DNA from *Escherichia coli* MG1655, *Lactobacillus plantarum* BCC9546, *Afifella marina* BCC40083, *Kineococcus gynurae* BCC26245, *Burkholderia tropica* BCC26066 or *Chryseobacterium indologenes* BCC19188 as a template with a taxon-specific primer pair (Table S3). A standard curve was constructed using 10-fold serial dilution of a plasmid DNA (from 10 to 10^8^ copy number) as a template in real-time PCR reaction. The real-time PCR was carried out in triplicates and a target copy number was calculated from the standard curve equation. Relative abundance for each target bacteria in each sample was determined by normalizing with the abundance of total bacteria in that sample, and percent relative abundance was determined within six target taxa.

### PCR Amplification of 16S rRNA Genes and Denaturing Gradient Gel Electrophoresis (DGGE)

PCR-DGGE profiles of intestine samples from four shrimp stages were performed according as previously described with some modifications [20], [75], [76]. Briefly, genomic DNA sample (final concentration of 1 ng/µl) from each library was used as a template for PCR reaction with primer pair 338F-GC clamps and 517R (Table S2). The PCR cycle parameters were 3 min initial denaturation at 94°C, 30 cycles of 30 sec at 94°C, 1 min at 55°C and 1.5 min at 72°C, and 10 min final extension at 72°C. The presence of a 200-bp fragment was confirmed on a 1.5% agarose gel electrophoresis. PCR-DGGE products were purified by illustra GFX PCR DNA and gel purification kit according to supplier’s manual (GE Healthcare, USA). Each PCR-DGGE product (600 ng) was loaded directly onto an 8% polyacrylamide gel with 40% to 60% denaturant vertical gradient. The in-house standard ladders were loaded as a control of DGGE analysis. The electrophoresis was performed at 80 V 60°C for 14 h using a DCode DGGE Electrophoresis System (Bio-Rad Laboratories, Inc., USA). After electrophoresis, the gels were stained using SYBR gold (Invitrogen, USA) and visualized under UV light gel doc system (Bio-Rad Laboratories, Inc.). All gel analyses were performed using InfoQuest™ Software (Bio-Rad Laboratories, Inc.). DGGE bands were excised and cloned to pGEM-T according to supplier’s instruction (Promega). Plasmid was extracted and submitted for sequencing. Sequence identity was determined by BLASTN in the GenBank database.

## Supporting Information

Figure S1 **Rarefaction analysis of 16S rRNA sequences from four black tiger shrimp growth stages: 15-day-old post-larva (PL15) and 1-, 2- and 3-month-old juveniles (J1, J2 and J3, respectively).** The operational taxonomic units (OTUs) were clustered at 0.03% dissimilarity level. Number of sequences refers to the number of pyrosequencing reads.
(TIF)

Table S1 **Taxonomical assignments of 16S rRNA sequences from pyrosequencing using the RDP classifier with a confidence threshold of 80%.**
(DOC)

Table S2 **Oligonucleotide used in this study.**
(DOC)

Table S3 **Bacteria-specific primers used for real-time PCR analysis.**
(DOC)

## References

[1] Hooper LV, Midtvedt T, Gordon JI (2002) How host-microbial interactions shape the nutrient environment of the mammalian intestine. Annu Rev Nutr 22: 283–307. 12055347 10.1146/annurev.nutr.22.011602.092259

[2] Li M, Wang B, Zhang M, Rantalainen M, Wang S, et al (2008) Symbiotic gut microbes modulate human metabolic phenotypes. PNAS 105: 2117–2122. 18252821 10.1073/pnas.0712038105 PMC2538887

[3] Rawls JF, Samuel BS, Gordon JI (2004) Gnotobiotic zebrafish reveal evolutionarily conserved responses to the gut microbiota. PNAS 101: 4596–4601. 15070763 10.1073/pnas.0400706101 PMC384792

[4] Brune A, Friedrich M (2000) Microecology of the termite gut: structure and function on a microscale. Curr Opin Microbiol 3: 263–269. 10851155 10.1016/s1369-5274(00)00087-4

[5] Harris JM (1993) The presence, nature, and role of gut microflora in aquatic invertebrates: A synthesis. Microbial Ecol 25: 195–231. 10.1007/BF00171889 24189919

[6] Xu J, Gordon JI (2003) Inaugural Article: Honor thy symbionts. PNAS 100: 10452–10459. 12923294 10.1073/pnas.1734063100 PMC193582

[7] Bates JM, Mittge E, Kuhlman J, Baden KN, Cheesman SE, et al (2006) Distinct signals from the microbiota promote different aspects of zebrafish gut differentiation. Dev Biol 297: 374–386. 16781702 10.1016/j.ydbio.2006.05.006

[8] Gomez-Gil B, Roque A, Turnbull JF (2000) The use and selection of probiotic bacteria for use in the culture of larval aquatic organisms. Aquaculture 191: 259–270.

[9] FAO (2003) Towards sustainable shrimp culture development: implementing the FAO code of conduct for responsible risheries (CCRF). Fisheries Department, Food and Agriculture Organization of the United Nations.

[10] Flegel TW (2012) Historic emergence, impact and current status of shrimp pathogens in Asia. J Invertebr Pathol 110: 166–173. 22429834 10.1016/j.jip.2012.03.004

[11] Tanticharoen M, Flegel TW, Meerod W, Grudloyma U, Pisamai N (2009) Aquacultural biotechnology in Thailand: the case of the shrimp industry. Int J Biotechnology 10: 588–603.

[12] Rengpipat S, Phianphak W, Piyatiratitivorakul S, Menasveta P (1998) Effects of a probiotic bacterium on black tiger shrimp *Penaeus monodon* survival and growth. Aquaculture 167: 301–313.

[13] Rengpipat S, Tunyanun A, Fast AW, Piyatiratitivorakul S, Menasveta P (2003) Enhanced growth and resistance to *Vibrio* challenge in pond-reared black tiger shrimp *Penaeus monodon* fed a *Bacillus* probiotic. Dis Aquat Organ 55: 169–173. 12911065 10.3354/dao055169

[14] Browdy CL (1998) Recent developments in penaeid broodstock and seed production technologies: improving the outlook for superior captive stocks. Aquaculture 164: 3–21.

[15] Lavilla-Pitogo CR, Leaño EM, Paner MG (1998) Mortalities of pond-cultured juvenile shrimp, *Penaeus monodon*, associated with dominance of luminescent vibrios in the rearing environment. Aquaculture 164: 337–349.

[16] Soto-Rodriguez SA, Gomez-Gil B, Lozano R, del Rio-Rodriguez R, Dieguez AL, et al (2012) Virulence of *Vibrio harveyi* responsible for the “Bright-red” Syndrome in the Pacific white shrimp *Litopenaeus vannamei* . J Invertebr Pathol 109: 307–317. 22306693 10.1016/j.jip.2012.01.006

[17] Jiravanichpaisal P, MIyazaki T, Limsuwan C (1994) Histopathology, Biochemistry, and Pathogenicity of *Vibrio harveyi* Infecting Black Tiger Prawn *Penaeus monodon* . J Aquat Anim Health 6: 27–35.

[18] Manefield M, Harris L, Rice SA, de Nys R, Kjelleberg S (2000) Inhibition of Luminescence and Virulence in the Black Tiger Prawn (*Penaeus monodon*) Pathogen *Vibrio harveyi* by Intercellular Signal Antagonists. Appl Environ Microbiol 66: 2079–2084. 10788385 10.1128/aem.66.5.2079-2084.2000 PMC101458

[19] Soonthornchai W, Rungrassamee W, Karoonuthaisiri N, Jarayabhand P, Klinbunga S, et al (2010) Expression of immune-related genes in the digestive organ of shrimp, *Penaeus monodon*, after an oral infection by *Vibrio harveyi* . Dev Comp Immunol 34: 19–28. 19646472 10.1016/j.dci.2009.07.007

[20] Chaiyapechara S, Rungrassamee W, Suriyachay I, Kuncharin Y, Klanchui A, et al. (2011) Bacterial Community Associated with the Intestinal Tract of *P. monodon* in Commercial Farms. Microb Ecol DOI: 10.1007/s00248-011-9936-2. 10.1007/s00248-011-9936-2 21915632

[21] Oxley AP, Shipton W, Owens L, McKay D (2002) Bacterial flora from the gut of the wild and cultured banana prawn, *Penaeus merguiensis* . J Appl Microbiol 93: 214–223. 12147069 10.1046/j.1365-2672.2002.01673.x

[22] Johnson CN, Barnes S, Ogle J, Grimes DJ, Chang Y-J, et al (2008) Microbial Community Analysis of Water, Foregut, and Hindgut during Growth of Pacific White Shrimp, *Litopenaeus vannamei*, in Closed-System Aquaculture. J World Aquacult Soc 39: 251–258.

[23] Margulies M, Egholm M, Altman WE, Attiya S, Bader JS, et al (2005) Genome sequencing in microfabricated high-density picolitre reactors. Nature 437: 376–380. 16056220 10.1038/nature03959 PMC1464427

[24] Costello EK, Lauber CL, Hamady M, Fierer N, Gordon JI, et al (2009) Bacterial community variation in human body habitats across space and time. Science 326: 1694–1697. 19892944 10.1126/science.1177486 PMC3602444

[25] Naqvi A, Rangwala H, Spear G, Gillevet P (2010) Analysis of multitag pyrosequence data from human cervical lavage samples. Chem Biodivers 7: 1076–1085. 20491067 10.1002/cbdv.200900321

[26] Ling Z, Kong J, Jia P, Wei C, Wang Y, et al (2010) Analysis of oral microbiota in children with dental caries by PCR-DGGE and barcoded pyrosequencing. Microb Ecol 60: 677–690. 20614117 10.1007/s00248-010-9712-8

[27] Roh SW, Kim KH, Nam YD, Chang HW, Park EJ, et al (2010) Investigation of archaeal and bacterial diversity in fermented seafood using barcoded pyrosequencing. ISME J 4: 1–16. 19587773 10.1038/ismej.2009.83

[28] Lauber CL, Hamady M, Knight R, Fierer N (2009) Pyrosequencing-Based Assessment of Soil pH as a Predictor of Soil Bacterial Community Structure at the Continental Scale. Appl Environ Microbiol 75: 5111–5120. 19502440 10.1128/AEM.00335-09 PMC2725504

[29] Roesch LF, Fulthorpe RR, Riva A, Casella G, Hadwin AK, et al (2007) Pyrosequencing enumerates and contrasts soil microbial diversity. ISME J 1: 283–290. 18043639 10.1038/ismej.2007.53 PMC2970868

[30] Jones RT, Robeson MS, Lauber CL, Hamady M, Knight R, et al (2009) A comprehensive survey of soil acidobacterial diversity using pyrosequencing and clone library analyses. ISME J 3: 442–453. 19129864 10.1038/ismej.2008.127 PMC2997719

[31] Parameswaran P, Jalili R, Tao L, Shokralla S, Gharizadeh B, et al (2007) A pyrosequencing-tailored nucleotide barcode design unveils opportunities for large-scale sample multiplexing. Nucleic Acids Res 35: e130. 17932070 10.1093/nar/gkm760 PMC2095802

[32] Hollister EB, Engledow AS, Hammett AJ, Provin TL, Wilkinson HH, et al (2010) Shifts in microbial community structure along an ecological gradient of hypersaline soils and sediments. ISME J 4: 829–838. 20130657 10.1038/ismej.2010.3

[33] Huber JA, Mark Welch DB, Morrison HG, Huse SM, Neal PR, et al (2007) Microbial population structures in the deep marine biosphere. Science 318: 97–100. 17916733 10.1126/science.1146689

[34] Sogin ML, Morrison HG, Huber JA, Mark Welch D, Huse SM, et al (2006) Microbial diversity in the deep sea and the underexplored "rare biosphere". PNAS 103: 12115–12120. 16880384 10.1073/pnas.0605127103 PMC1524930

[35] Zhang X, Yue S, Zhong H, Hua W, Chen R, et al (2011) A diverse bacterial community in an anoxic quinoline-degrading bioreactor determined by using pyrosequencing and clone library analysis. Appl Microbiol Biotechnol 91: 425–434. 21538111 10.1007/s00253-011-3296-1

[36] Kauserud H, Kumar S, Brysting AK, Norden J, Carlsen T (2011) High consistency between replicate 454 pyrosequencing analyses of ectomycorrhizal plant root samples. Mycorrhiza 10.1007/s00572-011-0403-1 [doi]. 10.1007/s00572-011-0403-1 21779811

[37] Telias A, White J, Pahl D, Ottesen A, Walsh C (2011) Bacterial community diversity and variation in spray water sources and the tomato fruit surface. BMC Microbiol 11: 81–93. 21510867 10.1186/1471-2180-11-81 PMC3108269

[38] Laycock G, Sait L, Inman C, Lewis M, Smidt H, et al (2012) A defined intestinal colonization microbiota for gnotobiotic pigs. Vet Immunol Immunopathol 149: 216–224. 22868203 10.1016/j.vetimm.2012.07.004

[39] Bäckhed F, Ley RE, Sonnenburg JL, Peterson DA, Gordon JI (2005) Host-bacterial mutualism in the human intestine. Science 307: 1915–1920. 15790844 10.1126/science.1104816

[40] Wang Q, Garrity GM, Tiedje JM, Cole JR (2007) Naive Bayesian classifier for rapid assignment of rRNA sequences into the new bacterial taxonomy. Appl Environ Microbiol 73: 5261–5267. 17586664 10.1128/AEM.00062-07 PMC1950982

[41] Good IJ (1953) The population frequencies of species and the estimation of population parameters. Biometrica 40: 237–264.

[42] Hill TC, Walsh KA, Harris JA, Moffett BF (2003) Using ecological diversity measures with bacterial communities. FEMS Microbiol Ecol 43: 1–11. 19719691 10.1111/j.1574-6941.2003.tb01040.x

[43] Dethlefsen L, Huse S, Sogin ML, Relman DA (2008) The Pervasive Effects of an Antibiotic on the Human Gut Microbiota, as Revealed by Deep 16S rRNA Sequencing. PLoS Biol 6: e280. 19018661 10.1371/journal.pbio.0060280 PMC2586385

[44] Andersson AF, Lindberg M, Jakobsson H, B√§ckhed F, Nyr√©n Pl, et al (2008) Comparative Analysis of Human Gut Microbiota by Barcoded Pyrosequencing. PLoS ONE 3: e2836. 18665274 10.1371/journal.pone.0002836 PMC2475661

[45] Gillilland MG, 3rd, Erb-Downward JR, Bassis CM, Shen MC, Toews GB, et al (2012) Ecological succession of bacterial communities during conventionalization of germ-free mice. Appl Environ Microbiol 78: 2359–2366. 22286988 10.1128/AEM.05239-11 PMC3302583

[46] Hughes JB, Hellmann JJ, Ricketts TH, Bohannan BJM (2002) Counting the Uncountable: Statistical Approaches to Estimating Microbial Diversity. Appl Environ Microbiol 68: 448. 10.1128/AEM.67.10.4399-4406.2001 PMC93182 11571135

[47] Roeselers G, Mittge EK, Stephens WZ, Parichy DM, Cavanaugh CM, et al (2011) Evidence for a core gut microbiota in the zebrafish. ISME J 5: 1595–1608. 21472014 10.1038/ismej.2011.38 PMC3176511

[48] Kim DH, Brunt J, Austin B (2007) Microbial diversity of intestinal contents and mucus in rainbow trout (Oncorhynchus mykiss). J Appl Microbiol 102: 1654–1664. 17578431 10.1111/j.1365-2672.2006.03185.x

[49] Navarrete P, Espejo RT, Romero J (2009) Molecular Analysis of Microbiota Along the Digestive Tract of Juvenile Atlantic Salmon (*Salmo salar* L.). Microb Ecol 57: 550–561. 18797955 10.1007/s00248-008-9448-x

[50] Thompson FL, Iida T, Swings J (2004) Biodiversity of Vibrios. Microbiol Mol Biol R 68: 403–431. 10.1128/MMBR.68.3.403-431.2004 PMC515257 15353563

[51] Liu H, Wang L, Liu M, Wang B, Jiang K, et al (2011) The intestinal microbial diversity in Chinese shrimp (*Fenneropenaeus chinensis*) as determined by PCR-DGGE and clone library analyses. Aquaculture 317: 32–36.

[52] Wilkenfeld JS, Lawrence AL, Kuban FD (1984) Survival, metamorphosis and growth of Penaeid shrimp larvae reared on a variety of algal and animal foods. J World Maricult Soc 15: 31–49.

[53] Chen H (1993) Recent Advances in Nutrition of *Penaeus monodon* . J World Aquacult Soc 24: 231–240.

[54] Chang ES (1995) Physiological and biochemical changes during the molt cycle in decapod crustaceans: an overview. J Exp Mar Biol Ecol 193: 1–14.

[55] Tkavc R, Ausec L, Oren A, Gunde-Cimerman N (2011) Bacteria associated with *Artemia* spp. along the salinity gradient of the solar salterns at Eilat (Israel). FEMS Microbiology Ecology 77: 310–321. 21492196 10.1111/j.1574-6941.2011.01112.x

[56] Hoj L, Bourne DG, Hall MR (2009) Localization, abundance and community structure of bacteria associated with *Artemia*: Effects of nauplii enrichment and antimicrobial treatment. Aquaculture 293: 278–285.

[57] Lopez-Torres MA, Lizarraga-Partida ML (2001) Bacteria isolated on TCBS media associated with hatched *Artemia* cysts of commercial brands. Aquaculture 194: 11–20.

[58] Leite AM, Mayo B, Rachid CT, Peixoto RS, Silva JT, et al (2012) Assessment of the microbial diversity of Brazilian kefir grains by PCR-DGGE and pyrosequencing analysis. Food Microbiol 31: 215–221. 22608226 10.1016/j.fm.2012.03.011

[59] Cleary DF, Smalla K, Mendonca-Hagler LC, Gomes NC (2012) Assessment of variation in bacterial composition among microhabitats in a mangrove environment using DGGE fingerprints and barcoded pyrosequencing. PLoS ONE 7: e29380. 22247774 10.1371/journal.pone.0029380 PMC3256149

[60] Ley RE, Peterson DA, Gordon JI (2006) Ecological and evolutionary forces shaping microbial diversity in the human intestine. Cell 124: 837–848. 16497592 10.1016/j.cell.2006.02.017

[61] Ribeiro F (2010) Development morphology and oncogeny of penaeid prawns postlarva; Alday-Sanz V, editor. Nottingham, UK: Nottingham University Press.

[62] Jiravanichpaisal P, Puanglarp N, Petkon S, Donnuea S, Söderhäll I, et al (2007) Expression of immune-related genes in larval stages of the giant tiger shrimp, *Penaeus monodon* . Fish & Shellfish Immunology 23: 815–824. 17490892 10.1016/j.fsi.2007.03.003

[63] Cahill MM (1990) Bacterial flora of fishes: A review. Microbiol Ecol 19: 21–41. 10.1007/BF02015051 24196252

[64] Lovett DL, Felder DL (1990) Ontogenetic Changes in Enzyme Distribution and Midgut Function in Developmental Stages of *Penaeus setiferus* (Crustacea, Decapoda, Penaeidae). Biol Bull 178: 160–174. 29314934 10.2307/1541974

[65] Palmer C, Bik EM, DiGiulio DB, Relman DA, Brown PO (2007) Development of the Human Infant Intestinal Microbiota. PLoS Biol 5: e177. 17594176 10.1371/journal.pbio.0050177 PMC1896187

[66] Perez T, Balcazar JL, Ruiz-Zarzuela I, Halaihel N, Vendrell D, et al (2010) Host-microbiota interactions within the fish intestinal ecosystem. Mucosal Immunol 3: 355–360. 20237466 10.1038/mi.2010.12

[67] Austin B, Zhang XH (2006) *Vibrio harveyi*: a significant pathogen of marine vertebrates and invertebrates. Lett Appl Microbiol 43: 119–124. 16869892 10.1111/j.1472-765X.2006.01989.x

[68] Balcazar JL, de Blas I, Ruiz-Zarzuela I, Cunningham D, Vendrell D, et al (2006) The role of probiotics in aquaculture. Vet Microbiol 114: 173–186. 16490324 10.1016/j.vetmic.2006.01.009

[69] Schloss PD, Westcott SL, Ryabin T, Hall JR, Hartmann M, et al (2009) Introducing mothur: Open-Source, Platform-Independent, Community-Supported Software for Describing and Comparing Microbial Communities. Appl Environ Microbiol 75: 7537–7541. 19801464 10.1128/AEM.01541-09 PMC2786419

[70] Edgar RC, Haas BJ, Clemente JC, Quince C, Knight R (2011) UCHIME improves sensitivity and speed of chimera detection. Bioinformatics doi10.1093/bioinformatics/btr381. 10.1093/bioinformatics/btr381 PMC3150044 21700674

[71] Kunin V, Engelbrektson A, Ochman H, Hugenholtz P (2010) Wrinkles in the rare biosphere: pyrosequencing errors can lead to artificial inflation of diversity estimates. Environ Microbiol 12: 118–123. 19725865 10.1111/j.1462-2920.2009.02051.x

[72] Huson DH, Auch AF, Qi J, Schuster SC (2007) MEGAN analysis of metagenomic data. Genome Res 17: 377–386. 17255551 10.1101/gr.5969107 PMC1800929

[73] Fierer N, Jackson JA, Vilgalys R, Jackson RB (2005) Assessment of soil microbial community structure by use of taxon-specific quantitative PCR assays. Appl Environ Microb 71: 4117–4120. 10.1128/AEM.71.7.4117-4120.2005 PMC1169028 16000830

[74] Bacchetti De Gregoris T, Aldred N, Clare AS, Burgess JG (2011) Improvement of phylum- and class-specific primers for real-time PCR quantification of bacterial taxa. J Microbiol Methods 86: 351–356. 21704084 10.1016/j.mimet.2011.06.010

[75] Muyzer G (1999) DGGE/TGGE a method for identifying genes from natural ecosystems. Curr Opin Microbiol 2: 317–322. 10383868 10.1016/S1369-5274(99)80055-1

[76] Muyzer G, de Waal EC, Uitterlinden AG (1993) Profiling of complex microbial populations by denaturing gradient gel electrophoresis analysis of polymerase chain reaction-amplified genes coding for 16S rRNA. Appl Environ Microbiol 59: 695–700. 7683183 10.1128/aem.59.3.695-700.1993 PMC202176

