# Supplementary figures and images for: Bacterial Population in Intestines of the Black Tiger Shrimp (Penaeus monodon) under Different Growth Stages
Source: PLoS One. 2013 Apr 5;8(4):e60802. doi: 10.1371/journal.pone.0060802 (PMC3618293; doi:10.1371/journal.pone.0060802)

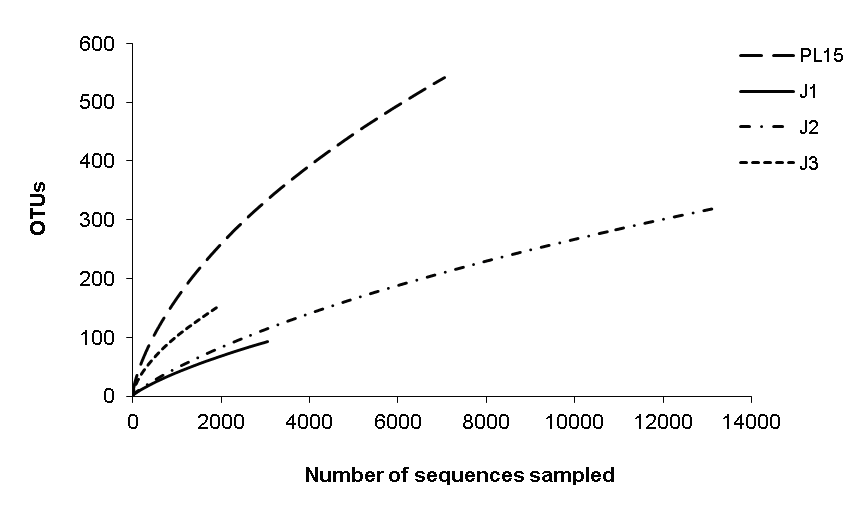

Supplement: Figure S1 — Rarefaction analysis of 16S rRNA sequences from four black tiger shrimp growth stages: 15-day-old post-larva (PL15) and 1-, 2- and 3-month-old juveniles (J1, J2 and J3, respectively). The operational taxonomic units (OTUs) were clustered at 0.03% dissimilarity level. Number of sequences refers to the number of pyrosequencing reads. (TIF) [file pone.0060802.s001.tif]
