# Supplementary material for: Bacterial Population in Intestines of the Black Tiger Shrimp (Penaeus monodon) under Different Growth Stages
Source: PLoS One. 2013 Apr 5;8(4):e60802. doi: 10.1371/journal.pone.0060802 (PMC3618293; doi:10.1371/journal.pone.0060802)
Supplement: Table S1 — Taxonomical assignments of 16S rRNA sequences from pyrosequencing using the RDP classifier with a confidence threshold of 80%. (DOC) [file pone.0060802.s002.doc]

**Table S1.** Taxonomical assignments of 16S rRNA sequences from pyrosequencing using the RDP classifier with a confidence threshold of 80%

| **Phylum** | **Classification** | **PL15** | **J1** | **J2** | **J3** |
| --- | --- | --- | --- | --- | --- |
| ***Actinobacteria*** | *Brevibacterium* | 2 |  | 1 |  |
|  | *Ilumatobacter* | 5 |  |  |  |
|  | *Rhodococcus* |  |  | 3 |  |
|  | Unclassified *Kineosporiaceae* |  | 3 |  |  |
|  | Unclassified *Microbacteriaceae* |  | 3 |  |  |
|  | Unclassified *Acidimicrobineae* | 7 |  |  |  |
|  | Unclassified *Micrococcineae* |  |  | 2 |  |
|  | Unclassified *Actinomycetales* | 2 | 1 |  |  |
|  | Unclassified *Actinobacteria* | 11 |  |  |  |
|  | **Total** | **27** | **7** | **6** | **0** |
|  |  |  |  |  |  |
| ***Bacteroidetes*** | *Aquimarina* | 5 |  |  |  |
|  | *Chryseobacterium* | 2 |  |  |  |
|  | *Cloacibacterium* | 16 |  |  |  |
|  | *Crocinitomix* | 3 |  |  |  |
|  | *Flavobacterium* | 4 | 1 |  |  |
|  | *Gilvibacter* | 5 |  |  |  |
|  | *Lishizhenia* | 3 |  |  |  |
|  | *Meridianimaribacter* | 3 |  |  | 3 |
|  | *Muricauda* |  |  |  | 1 |
|  | *Nubsella* | 4 |  |  |  |
|  | *Owenweeksia* |  |  |  | 3 |
|  | *Pedobacter* | 24 |  |  |  |
|  | *Sandarakinotalea* | 3 |  |  |  |
|  | *Tenacibaculum* | 8 |  |  |  |
|  | *Terrimonas* | 1 |  |  |  |
|  | Unclassified *Chitinophagaceae* | 3 |  | 2 |  |
|  | Unclassified *Cryomorphaceae* | 3 |  |  |  |
|  | Unclassified *Flavobacteriaceae* | 161 | 3 | 4 | 11 |
|  | Unclassified *Saprospiraceae* | 2 |  |  |  |
|  | Unclassified *Sphingobacteriaceae* | 3 | 1 |  |  |
|  | Unclassified *Flavobacteriales* | 72 |  |  |  |
|  | Unclassified *Sphingobacteriales* |  | 2 |  |  |
|  | Unclassified *Bacteroidetes* | 35 | 8 |  |  |
|  | **Total** | **360** | **15** | **6** | **18** |
|  |  |  |  |  |  |
| ***Firmicutes*** |  |  |  |  |  |
|  | *Bacillus* | 2 | 4 |  | 1 |
|  | *Clostridium sensu stricto* | 1 |  |  |  |
|  | *Exiguobacterium* | 9 |  |  |  |
|  | *Fusibacter* | 32 |  | 6 |  |
|  | *Lactobacillus* | 52 | 1 | 1 |  |
|  | *Lactococcus* | 7 |  | 1 |  |
|  | *Streptococcus* | 5 |  | 2 |  |
|  | *Weissella* | 1 |  |  |  |
|  | Unclassified *Lactobacillaceae* | 2 |  |  |  |
|  | Unclassified *Bacillales* | 7 | 1 |  |  |
|  | Unclassified *Clostridiales* | 25 |  | 1 |  |
|  | Unclassified *Firmicutes* | 10 |  | 20 |  |
|  | **Total** | **153** | **6** | **31** | **1** |
|  |  |  |  |  |  |
| ***Fusobacteria*** | *Fusobacterium* | 1 |  |  |  |
|  | Unclassified *Fusobacteriaceae* | 19 |  |  |  |
|  | Unclassified *Fusobacteriales* | 1 |  |  |  |
|  | **Total** | **21** | **0** | **0** | **0** |
|  |  |  |  |  |  |
| ***Proteobacteria*** |  |  |  |  |  |
| ***α-Proteobacteria*** | *Altererythrobacter* | 1 |  |  |  |
|  | *Bosea* | 2 |  | 1 |  |
|  | *Bradyrhizobium* | 4 |  | 4 |  |
|  | *Brevundimonas* | 10 | 1 | 1 |  |
|  | *Brucella* | 5 |  | 7 |  |
|  | *Caulobacter* | 3 |  | 1 |  |
|  | *Cohaesibacter* | 1 |  | 1 |  |
|  | *Erythrobacter* | 1 |  |  |  |
|  | *Filomicrobium* | 1 |  |  |  |
|  | *Jannaschia* | 3 |  |  |  |
|  | *Kiloniella* |  |  |  | 1 |
|  | *Labrenzia* |  |  |  | 63 |
|  | *Litoreibacter* | 2 |  |  |  |
|  | *Methylobacterium* | 6 | 1 |  |  |
|  | *Nautella* | 1 |  |  |  |
|  | *Novosphingobium* | 10 |  |  |  |
|  | *Oceanicola* |  |  |  | 1 |
|  | *Phenylobacterium* | 6 |  | 5 |  |
|  | *Pseudoruegeria* | 2 |  |  |  |
|  | *Rhodobacter* |  | 2 |  |  |
|  | *Roseibium* |  |  |  | 2 |
|  | *Roseovarius* | 6 |  |  |  |
|  | *Ruegeria* | 3 |  |  | 4 |
|  | *Silicibacter* |  |  |  | 43 |
|  | *Shimia* | 13 |  |  |  |
|  | *Sphingobium* | 18 |  |  |  |
|  | *Sphingomonas* | 12 |  | 7 |  |
|  | *Thalassobius* | 57 |  |  |  |
|  | *Xanthobacter* | 3 |  |  |  |
|  | *Zhangella* | 1 |  |  | 2 |
|  | Unclassified *Brucellaceae* |  |  | 1 |  |
|  | Unclassified *Hyphomicrobiaceae* |  |  |  | 2 |
|  | Unclassified *Phyllobacteriaceae* | 4 |  |  |  |
|  | Unclassified *Rhodobacteraceae* | 249 | 9 | 2 | 59 |
|  | Unclassified *Sphingomonadaceae* | 39 |  |  |  |
|  | Unclassified *Sphingomonadales* | 10 |  | 3 |  |
|  | Unclassified *Alphaproteobacteria* | 5 | 2 | 4 | 1 |
|  | **Total** | **478** | **15** | **37** | **178** |
|  |  |  |  |  |  |
| ***β-Proteobacteria*** | *Achromobacter* | 2 |  | 1 |  |
|  | *Acidovorax* | 5 |  |  |  |
|  | *Aquabacterium* | 12 |  |  |  |
|  | *Azospira* | 8 |  |  |  |
|  | *Burkholderia* | 1 |  |  |  |
|  | *Comamonas* | 17 |  |  |  |
|  | *Diaphorobacter* | 2 |  |  |  |
|  | *Herbaspirillum* | 1 |  |  |  |
|  | *Janthinobacterium* | 14 |  | 2 |  |
|  | *Methylotenera* | 1 |  |  |  |
|  | *Pelomonas* | 1 |  |  |  |
|  | *Zoogloea* | 1 |  |  |  |
|  | *Undibacterium* | 13 |  |  |  |
|  | Unclassified *Alcaligenaceae* |  |  | 1 |  |
|  | Unclassified *Burkholderiales_incertae_sedis* | 2 |  |  |  |
|  | Unclassified *Comamonadaceae* | 3 | 1 | 1 |  |
|  | Unclassified *Methylophilaceae* | 2 |  |  |  |
|  | Unclassified *Rhodocyclaceae* | 3 |  |  |  |
|  | Unclassified *Burkholderiales* | 5 |  |  |  |
|  | **Total** | **93** | **1** | **5** | **0** |
|  |  |  |  |  |  |
| ***δ-Proteobacteria*** |  |  |  |  |  |
|  | *Bacteriovorax* | 3 |  |  |  |
|  | *Desulfosarcina* | 7 |  |  |  |
|  | *Desulfovibrio* | 2 | 1 |  |  |
|  | Unclassified *Deltaproteobacteria* | 2 |  |  |  |
|  | **Total** | **14** | **1** | **0** | **0** |
|  |  |  |  |  |  |
| ***ε-Proteobacteria*** |  |  |  |  |  |
|  | *Arcobacter* | 15 |  |  |  |
|  | **Total** | **15** | **0** | **0** | **0** |
|  |  |  |  |  |  |
| ***γ-Proteobacteria*** |  |  |  |  |  |
|  | *Acinetobacter* | 73 |  | 2 |  |
|  | *Aeromonas* |  | 3 |  |  |
|  | *Allomonas* | 6 |  | 1 |  |
|  | *Alteromonas* | 4 |  |  |  |
|  | *Amphritea* | 2 |  |  |  |
|  | *Bowmanella* |  |  |  | 3 |
|  | *Escherichia/Shigella* | 2 | 5 | 4 |  |
|  | *Ferrimonas* | 1 |  |  |  |
|  | *Francisella* |  |  |  | 4 |
|  | *Grimontia* | 1 |  |  |  |
|  | *Haemophilus* | 6 |  |  |  |
|  | *Halomonas* |  |  |  | 2 |
|  | *Legionella* | 3 |  |  |  |
|  | *Listonella* | 3 | 36 | 201 | 12 |
|  | *Marinicella* | 7 |  |  |  |
|  | *Marinomonas* |  |  |  | 3 |
|  | *Methylophaga* | 6 |  |  |  |
|  | *Photobacterium* | 2,424 | 1 | 7 | 1 |
|  | *Pseudoalteromonas* | 7 |  |  | 10 |
|  | *Pseudomonas* | 62 | 1 | 12 | 3 |
|  | *Psychrobacter* | 3 |  |  |  |
|  | *Serratia* | 1 |  | 1 |  |
|  | *Shewanella* | 1 |  | 8 |  |
|  | *Stenotrophomonas* | 2 |  |  |  |
|  | *Proteus* |  | 1 |  |  |
|  | *Thalassomonas* | 3 |  |  |  |
|  | *Vibrio* | 429 | 600 | 1,938 | 622 |
|  | *Yersinia* | 1 |  |  |  |
|  | Unclassified *Aeromonadaceae* |  |  | 1 |  |
|  | Unclassified *Alteromonadaceae* | 2 |  |  |  |
|  | Unclassified *Alteromonadales* | 1 | 2 | 25 |  |
|  | Unclassified *Enterobacteriaceae* | 1 |  |  |  |
|  | Unclassified *Methylococcaceae* |  |  |  | 1 |
|  | Unclassified *Moraxellaceae* | 1 |  |  |  |
|  | Unclassified *Pasteurellaceae* | 3 |  |  |  |
|  | Unclassified *Pseudomonadaceae* | 2 |  | 2 | 6 |
|  | Unclassified *Vibrionaceae* | 1,885 | 2,361 | 10,742 | 1,025 |
|  | Unclassified *Gammaproteobacteria* | 742 |  | 21 |  |
|  | **Total** | **5,684** | **3,010** | **12,965** | **1,692** |
|  |  |  |  |  |  |
| **Unclassified *Proteobacteria*** |  | 9 |  | 1 |  |
|  | **Total** | **9** | **0** | **1** | **0** |
|  |  |  |  |  |  |
| ***Spirochaetes*** | *Spirochaeta* |  |  | 4 |  |
|  | **Total** | **0** | **0** | **4** | **0** |
|  |  |  |  |  |  |
| **Unclassified Bacteria** |  | 191 |  | 75 | 1 |
|  | **Total** | **191** | **0** | **75** | **1** |
| **Total sequences** |  | **7,045** | **3,055** | **13,130** | **1,890** |
