# Supplementary material for: Bacterial Population in Intestines of the Black Tiger Shrimp (Penaeus monodon) under Different Growth Stages
Source: PLoS One. 2013 Apr 5;8(4):e60802. doi: 10.1371/journal.pone.0060802 (PMC3618293; doi:10.1371/journal.pone.0060802)
Supplement: Table S2 — Oligonucleotide used in this study. (DOC) [file pone.0060802.s003.doc]

**Table S2.** Oligonucleotide used in this study.

| **Name** | **Sequence 5’ to 3’**^1^ |
| --- | --- |
| 338F-PL15 | **ATCAGTCG**ACTCCTACGGGAGGCAGCAG |
| 786R-PL15 | **ATCAGTCG**CTACCAGGGTATCTAATC |
| 338F-J1 | **TACTACGC**ACTCCTACGGGAGGCAGCAG |
| 786R-J1 | **TACTACGC**CTACCAGGGTATCTAATC |
| 338F-J2 | **AGCAGAGC**ACTCCTACGGGAGGCAGCAG |
| 786R-J2 | **AGCAGAGC**CTACCAGGGTATCTAATC |
| 338F-J3 | **TCACTGTC**ACTCCTACGGGAGGCAGCAG |
| 786R-J3 | **TCACTGTC**CTACCAGGGTATCTAATC |
| 5’ overhung adaptor A | CCATCTCATCCCTGCGTGTCCCATCTGTTCCCTCCCTGTCTCAG |
| 5’ overhung adaptor B | CCTATCCCCTGTGTGCCTTGCCTATCCCCTGTTGCGTGTCTCAG |
| 338GC-F | cgcccgccgcgcgcggcgggcggggcgggggcacggggggactcctacgggaggca |
| 517R | 5′ ATTACCGCGGCTGCTGG 3′ |

^1^ The underline indicates barcoded sequence region.
