# Supplementary material for: Bacterial Population in Intestines of the Black Tiger Shrimp (Penaeus monodon) under Different Growth Stages
Source: PLoS One. 2013 Apr 5;8(4):e60802. doi: 10.1371/journal.pone.0060802 (PMC3618293; doi:10.1371/journal.pone.0060802)
Supplement: Table S3 — Bacteria-specific primers used for real-time PCR analysis. (DOC) [file pone.0060802.s004.doc]

**Table S3**. Bacteria-specific primers used for realtime PCR analysis.

| **Target group** | **Name** | **Sequence 5’ to 3’** | **Reference** |
| --- | --- | --- | --- |
| All bacteria | Eub338 | ACTCCTACGGGAGGCAGCAG | [1] |
|  | Eub518 | ATTACCGCGGCTGCTGG |  |
| Firmicutes | Lgc353 | GCAGTAGGGAATCTTCCG |  |
| Betaproteobacteria | Bet680 | TCACTGCTACACGYG |  |
| Gammaproteobacteria | 1080γF | TCGTCAGCTCGTGTYGTGA | [2] |
|  | γ1202R | CGTAAGGGCCATGATG |  |
| Alphaproteobacteria | α682F | CIAGTGTAGAGGTGAAATT |  |
|  | 908αR | CCCCGTCAATTCCTTTGAGTT |  |
| Actinobacteria | Act920F3 | TACGGCCGCAAGGCTA |  |
|  | Act1200R | TCRTCCCCACCTTCCTCCG |  |
| Bacteroidetes | 798cfbF | CRAACAGGATTAGATACCCT |  |
|  | Cfb967R | GGTAAGGTTCCTCGCGTAT |  |

**References**

1. Fierer N, Jackson JA, Vilgalys R, Jackson RB (2005) Assessment of soil microbial community structure by use of taxon-specific quantitative PCR assays. Appl Environ Microb 71: 4117-4120.

2. Bacchetti De Gregoris T, Aldred N, Clare AS, Burgess JG (2011) Improvement of phylum- and class-specific primers for real-time PCR quantification of bacterial taxa. J Microbiol Methods 86: 351-356.
